# Posterior reversible encephalopathy syndrome associated with dengue fever induced intrauterine death: A case report

**DOI:** 10.1002/ccr3.8575

**Published:** 2024-03-04

**Authors:** Himel Kumar Biswas, Khadija Tanbil Ishaque Ibu, Rama Biswas, Md Nasir Uddin Ahmed

**Affiliations:** ^1^ Department of Neurology Square Hospital Dhaka Bangladesh; ^2^ Department of Critical Care Medicine Anwer Khan Modern Medical College Hospital Dhaka Bangladesh

**Keywords:** dengue, dengue expanded syndrome, intrauterine death, neurology, posterior reversible encephalopathy syndrome

## Abstract

In recent years dengue fever has become a major health concern specifically due to its diverse presentation and adverse outcome. Progression from mild febrile illness to a severe systemic illness may occur in dengue fever including neurological disorder. Here, we report an unusual and rare case of a 20‐year‐old mother who developed posterior reversible encephalopathy syndrome (PRES) following dengue fever induced intrauterine death and septic shock.

## Graphical abstract

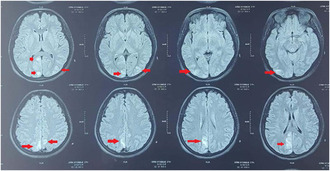

## 1 INTRODUCTION

Unusual complications of dengue are becoming more common nowadays, however, these complications are not always recognized or understood due to a lack of awareness of these adverse outcomes. Not many studies have been undertaken to document all the complications of dengue expanded syndrome, but so far it is seen to affect the major organs like liver, lung, kidney, heart, and brain.^1^ Moreover, neurological manifestations of dengue fever are extremely rare where most reported cases are intracranial hemorrhage, encephalitis, and acute disseminated encephalomyelitis (ADEM). In a study conducted between 2014 and 2019 showed only 2.64% of 5821 dengue patients were affected with neurological manifestations.^2^ Furthermore, another study found the incidence rate ranging from 0.5% to 20% in recent years.^3^ We are presenting a case of posterior reversible encephalopathy syndrome (PRES) which is most likely the rarest presentation of dengue expanded syndrome identified and diagnosed at a tertiary level hospital. PRES manifests' as characteristic neuroimaging findings along with common clinical symptoms of headache, visual loss, seizure, and raised blood pressure.^4^ Although PRES has not frequently been reported in dengue, they may have some similarity in their pathogenesis where both are triggered by identical endothelial dysfunction.^5^

Radiologically, PRES is defined by bilateral white matter augmentation in T2 signal intensity on MRI, which is typically noticed in the posterior region of the hemispheres. However, the white matter vasogenic edema which is supposed to be found to the posterior cerebrum is not always confined to that area, sometimes thalamus and anterior circulation are also affected. Although, this usually normalizes in a few weeks by resolving underlying risk factors and removal of causative agents, can lead to much deleterious effects if it remains untreated.^6^ So, here we can include PRES as a manifestation of dengue expanded syndrome which may remain undiagnosed and should be considered in dengue patients with neurological symptoms.

## 2 CASE REPORT

A 20‐year‐old 37 weeks pregnant lady (Parity‐0 and Gravida‐1) was admitted to the hospital with fever and headache for last 8 days with less fetal movement and severe abdominal pain. On presentation, her pulse was122 bpm, blood pressure 110/70 mm of Hg, temperature 102°F and oxygen saturation (SpO_2_) was of 96% on room air, while fetal heart sound was absent in auscultation with unremarkable systemic examination. She was found positive for dengue nonstructural protein 1 (NS1) antigen and immunoglobulin M but negative for dengue immunoglobulin G. Unfortunately, intrauterine death (IUD) was confirmed by ultrasound. She became disoriented and confused along with vision loss following normal vaginal delivery of the IUD baby on the next day. She had convulsion for a single episode which was treated promptly with benzodiazepine and was started on intravenous levetiracetum. Patient was needed oxygen support 10 L/min via Hudson mask and she was shifted to intensive care unit (ICU) immediately. Her consciousness level continued to deteriorate (Glasgow Coma Scale‐E3V3M4) and became hemodynamically unstable‐blood pressure became non‐recordable, pulse 138 bpm. Her blood reports showed neutrophilic leukocytosis with thrombocytopenia, significantly raised liver enzymes, PT, APTT, and D‐Dimer with altered renal function test (Table 1). Initially, she was diagnosed as a case of dengue expanded syndrome with disseminated intravascular coagulation (DIC) with septic shock. Treatment was continued with intravenous (I/V) antibiotics, adequate I/V fluid, inotropes support, and other supportive management according to standard protocol. Four units of whole blood and single unit aphaeretic platelet were transfused.

**TABLE 1:** Results of laboratory investigations.

Investigations	Value	Normal range
Hemoglobin (gm/dL)	7.0	12–17
Platelet (×10^9^/L)	92	150–410
Total count (×10^9^/L)	33	4–10
ALP (IU/L)	220	30–150
ALT (IU/L)	1442	<35
AST (IU/L)	9698	<35
Sodium (mmol/L)	130	135.0–145.0
Potassium (mmol/L)	5.2	3.5–5.0
Calcium (mg/dL)	7.8	8.5–10.5
Ammonia (μg/dL)	95	15–60
Magnesium (mg/dL)	3.2	1.6–2.3
Creatinine (mg/dL)	1.0	0.52–1.04
Urea (mg/dL)	26	15–36
Dengue NS1	Positive	–
PT (s)	30	10.7–13.6
APTT (s)	46	23.0–29.9
CRP (mg/dL)	34	<10
Procalcitonin (mg/dL)	8.67	≤0.046
Lactate (mmol/L)	13.28	<2.0
D‐Dimer (mg/L)	6.0	0.50
Troponin‐I (ng/mL)	33	<0.034

**FIGURE 1:**
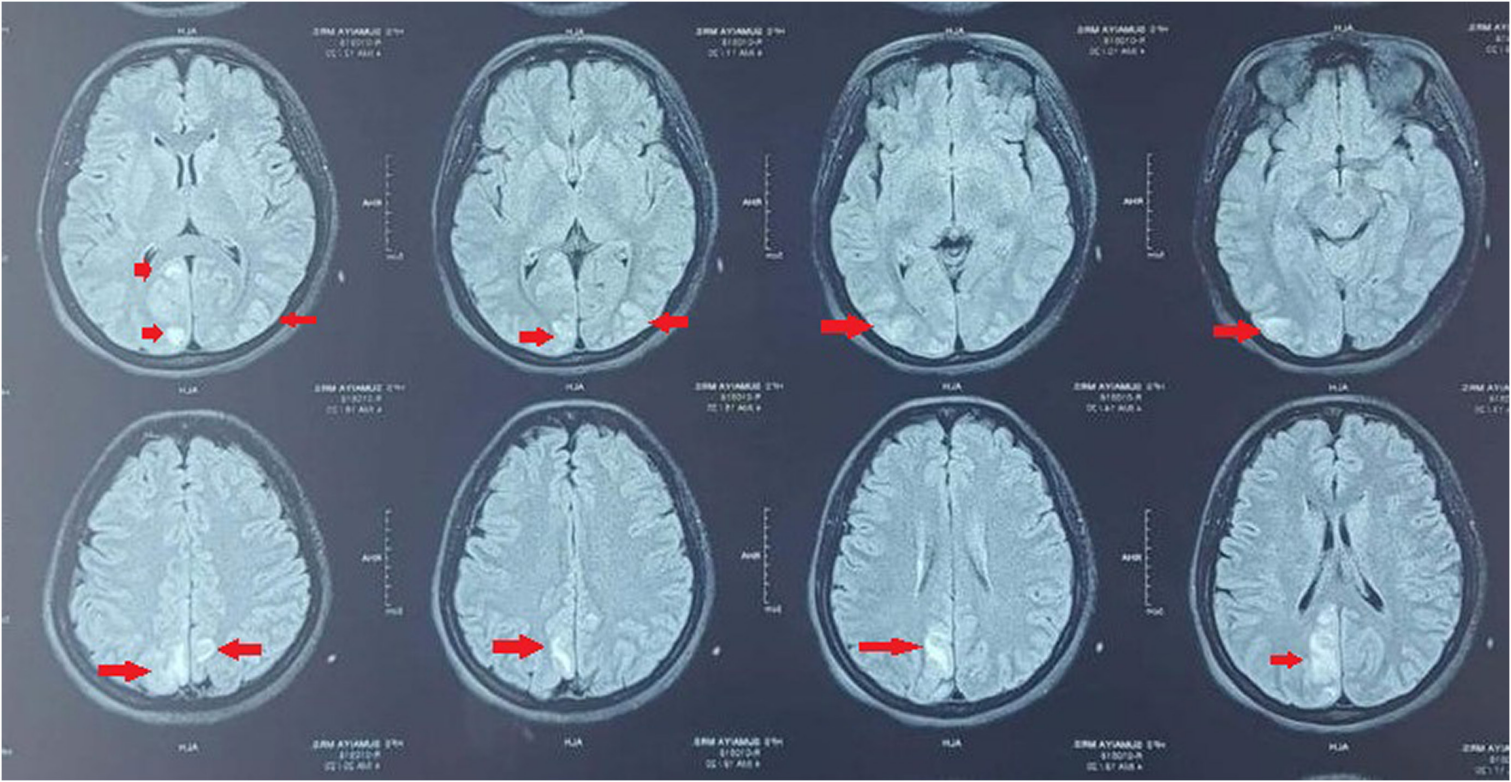
T2‐FLAIR hyperintense areas with mild restricted diffusion are seen in both posterior parietal and occipital regions. Similar signal areas are also seen in posterior‐inferior aspects of cerebellum.

On the following date, CE‐MRI showed cortico‐subcortical T2 weighted fluid attenuated inversion recovery (FLAIR) hyperintense areas with mild restricted diffusion in both posterior parietal and occipital regions, also postero‐inferior aspect of cerebellum with similar intense areas which suggestive of PRES (Figure 1). Her condition was very critical and unfortunately, due to financial issue patient's family members couldn't afford to keep her in ICU and was discharged against medical advice. Day after discharge she was passed away according to her family members.

## 3 DISCUSSION

A specific clinicoradiological illness known as PRES is characterized by reversible brain vasogenic edema along with altered consciousness, seizures, headache, and visual problems.^4^ Acute hypertension, hypertensive disorders during pregnancy, autoimmune disorders (such as systemic sclerosis and systemic lupus erythematosus), immunosuppressive medications, renal failure, cytotoxic drugs, and infection are some of the causes. Dengue virus and other infectious etiologies, including viruses, sepsis, have all been linked to PRES.^5^, ^7^

Mild to severe febrile illness with systemic complications such as plasma leakage, bleeding, shock, and myocarditis with some neurotropic disorder may happen in some patients, though neurological complications are rare in dengue fever.^5^ In addition, ADEM, dengue encephalopathy, meningitis, Guillain–Barre syndrome (GBS) were found in recent outbreak of dengue.^8^ But dengue in a pregnant mother can cause some unusual manifestations in both mother and baby. Pregnancy‐related dengue virus infection often causes miscarriage, IUD, and secondary neonatal damage, which includes premature birth and vertical viral transmission.^9^ In a study conducted by Carles et al. in French Guiana involving 38 pregnant mothers with confirmed dengue, utero fetal death was 1.9%.^10^ In another study by Gehlot et al. in India showed 4% of IUD in dengue positive pregnant women^11^ with other complications.

Since the exact etiology of dengue following PRES is unknown, it is thought to be associated with endothelial dysfunction and a failure to cerebral autoregulation, which results in vasogenic edema and reduced cerebral blood flow.^5^ Although cerebral edema and disease severity may correlate with high CSF protein, without any cellular reaction CSF pressure and protein may elevate more than 100 mg/dL.^12^ Moreover, Increase in vascular permeability due to release of inflammatory cytokines resulted in interstitial brain edema.^13^, ^14^, ^15^ The characteristic MRI finding of vasogenic edema involving parieto‐occipital region of both cerebral hemispheres are essential for diagnosis. Frequently the subcortical white matter is involved. The holohemispheric watershed pattern, superior frontal sulcus pattern, and dominant parieto‐occipital pattern are the three main magnetic resonance imaging (MRI) brain patterns that have been identified till now.^16^, ^17^, ^18^ In terms of etiology, our patient did not have any strongly related illnesses such as chronic kidney disease (CKD), systemic lupus erythematosus (SLE), hypertensive emergency, and immunosuppressive medications or use of chemotherapeutic agents like cyclosporine, tacrolimus, vincristine, or alpha‐interferon.^19^, ^20^ Although the mainstay of treatment is supportive, any causative agent should be identified and removed to improve outcome. Management of PRES differs from dengue encephalitis and ADEM. Because, ADEM treatment requires potential steroid therapy whereas PRES treatment requires supportive symptomatic management only.^17^

## 4 CONCLUSION

The current concept of dengue fever is changing every day and it's becoming more difficult to treat a dengue patient because of emerging unexpected complications. While treating a severely ill dengue patient, we should also consider neurological issues which can be reversed with proper treatment. This mother was very unfortunate who was complicated by IUD of her child, could have a better outcome if could seek early medical advice. PRES may remain unrecognized in dengue patients due to overlapping symptoms and should take into account and check by doing MRI brain.

## AUTHOR CONTRIBUTIONS

**Himel Kumar Biswas:** Conceptualization; formal analysis; investigation; writing – original draft. **Khadija Tanbil Ishaque Ibu:** Investigation; resources; validation; writing – review and editing. **Rama Biswas:** Supervision; visualization. **Md Nasir Uddin Ahmed:** Resources.

## FUNDING INFORMATION

This study received no financing from any governmental, corporate, or non‐profit organization.

## CONFLICT OF INTEREST STATEMENT

The authors state that they have no known conflicting financial interests or personal relationships that may be seen as having influenced the work described in this study.

## ETHICS STATEMENT

As per our institutional ethics committee guidelines, we do not require ethics approval for publishing case reports.

## CONSENT

Written informed consent was obtained from the patient's husband to publish this report including clinical facts and imaging in this article in accordance with the journal's patient consent policy.

## Data Availability

All the required information is available in the manuscript itself.
